# Clinical Profile of Diabetes Mellitus in Young Patients Admitted at BP Koirala Institute of Health Sciences: A Cross‐Sectional Study

**DOI:** 10.1002/hsr2.72206

**Published:** 2026-04-15

**Authors:** Dinesh Upreti, Subarna Giri, Smile Kharel, Laxmi Shrestha, Robin Maskey

**Affiliations:** ^1^ Maharajgunj Medical Campus Tribhuvan University Institute of Medicine Nepal; ^2^ Universal College of Medical Sciences Nepal; ^3^ Lumbini Medical College Nepal; ^4^ Department of Internal Medicine BP Koirala Institute of Health Sciences Nepal

**Keywords:** diabetes in young, diabetes mellitus, insulin‐dependent, Nepal

## Abstract

**Background and Aims:**

Incidence of diabetes is increasing at a younger age, causing a significant economic burden, especially in developing countries. This study was conducted to find the sociodemographic distribution of diabetes in young adults, along with clinical, laboratory, and treatment strategies used.

**Methods:**

A cross‐sectional observational study was conducted including all young patients (20–39 years) diagnosed with diabetes in a tertiary center of Nepal over 1 year duration. Patients with secondary diabetes, such as gestational diabetes, were excluded. Sociodemographic, clinical, relevant laboratory parameters, and treatment‐related data were collected and analyzed using SPSS.

**Results:**

There were a total of 133 patients, with a mean age of 31.40 ± 5.4 years. 82% were symptomatic at diagnosis, with polyuria as the most common symptom. The mean duration of diabetes was 5.4 ± 0.4 years, and 24% of the patients were insulin dependent. Family history of diabetes was present in 46 (34.60%). A total of 69 (52%) patients had some sort of complications, with the majority showing neuropathies. 31 (23%) developed ketoacidosis.

**Conclusions:**

Based on this study, we discovered that the presence of ketoacidosis is associated with patients with diabetes in the lower age group, relatively lower BMI, and heralds the T1DM group on the basis of clinical profile.

## 1 Introduction

Young adulthood stretches from 20 to 40 years of age according to Erikson's staging [1]. The incidence of diabetes mellitus is starting to rise at a younger age [2]. The global burden of Type 1 Diabetes mellitus (T1DM) increased from 153.42 per 100,000 population in 1990 to 195.75 per 100,000 in 2019. A rapid increase in incidence and prevalence of T1 DM was observed in young adults 20–24 years [3]. A study reported 4.8% annual increase in the incidence of type 2 diabetes mellitus (T2DM) from 2002 to 2003 to 2011–2012 in adolescents aged 10–19 years [4]. There is a varying presentation of diabetes in young adults. It possesses a significant economic burden on health resources worldwide, especially in developing countries. The types of diabetes in the youth population are classified into classical T1DM, classical T2DM, ketosis‐resistant youth diabetes, pancreatic diabetes, or maturity‐onset diabetes mellitus [5]. The diagnosis and classification of diabetes are based on the clinical and immunological profile of the patients [5]. A MEDI study (Multicenter Survey of Early Onset Diabetes in India), a large multicenter study, showed that T1DM is the commonest form of diabetes in childhood [6]. The prevalence of diabetes mellitus in productive age (18–55 years) urban Indonesians was 4.6%, consisting of 1.1% previously diagnosed diabetes mellitus and 3.5% undiagnosed diabetes mellitus [2]. According to a nationally representative study, the prevalence of diabetes in Nepal was 8.5%. The proportion of diabetes in the population of the age group 20–39 years was 3% [7].

A systematic review summarized the prevalence of type 2 diabetes in Nepal to be 8.4% [8]. One of the studies showed the increasing prevalence of T2DM from 584.81 per 1000 population in the age group 15–19 years to 22,345.74 per 1000 population of age ≥ 70 years [9].

Despite genetic and ethnic similarities with neighboring countries, differences in healthcare access, nutritional status, socioeconomic factors, urbanization patterns, and healthcare‐seeking behavior may significantly influence the prevalence and clinical profile of diabetes among the young population in Nepal. There is a dearth of information about various types of diabetes mellitus and their characteristics in Nepal, focusing on young patients. Most of the study has focused on T2DM, which is more common in the older age group. So, this study was done to find out the demographic, clinical, and laboratory picture of diabetes in the younger age group (20–39 years). Secondary objectives include identifying outcomes, complications, and different modalities of treatment.

## 2 Materials and Methods

### 2.1 Study Design

This is a single Institution based, observational, cross‐sectional study.

### 2.2 Study Site and Duration

This study was carried out in the Department of Internal Medicine, BP Koirala Institute for Health Sciences for a period of 1 year, 2017–2018 (outpatient and inpatient basis) after ethical clearance from the Institute Research Council.

### 2.3 Inclusion Criteria

1. Patient diagnosed with Diabetes mellitus
2. Young adults between 20 and 39 years of age
3. Those providing informed written consent

### 2.4 Exclusion Criteria

Patient with secondary diabetes, Gestational Diabetes Mellitus, New onset Diabetes associated with Transplant, Drug‐induced.

### 2.5 Study Setting

The study was done in a hospital setting, enrollment of patients was done in the Outpatient department and the Ward of Internal Medicine, BPKIHS.

### 2.6 Sample Size Calculation

Sample size (*n*) was calculated by the formula given below to ensure adequate representation of young adults with diabetes presenting to a tertiary center during the study period:

$$n=Z\alpha 2\times p\times q/d^{2}$$

Where,

*n* = sample size,

*Zα* = *Z* statistic for a level of confidence. At 95% confidence interval where alpha error =5%, *Zα* = 1.96

*p* = Expected prevalence = 9.5% [8]

*q* = 1−*p* = 91.5%

*d* = Precision = 10% of *p,* i.e., consider 90% power

**On calculation:**

*n* = 133

### 2.7 Ethical Approval and Patient Consent

Ethical clearance was obtained from the Institutional Review Committee and Protocol Evaluation Committee of BPKIHS (311/073/074‐IRC). Participation of patients was ensured using an informed consent form.

### 2.8 Study Tools and Techniques

The data collection tool was a structured questionnaire developed by the investigators based on prior studies and clinical experience. Formal psychometric validation was not performed, which may limit reproducibility. The socio‐demographic characteristics & other relevant data were taken from face‐to‐face interviews and paper pencil questionnaire, eliciting data on age, gender, occupation, address (Terai/Hill/Mountain region), ethnicity, marital status, and education (With formal education/without formal education). Personal history included a history of smoking and alcohol consumption (social drinker/regular consumer). Detailed clinical examination, blood sugar profile (fasting, post‐prandial, and HbA1c, insulin, and C‐peptide level), and relevant investigation as per the standard protocol were done. ssCriteria for diagnosis of diabetes were as per standard American Diabetes Association (ADA) guidelines [10]. A questionnaire including age, gender, symptoms of diabetes, beginning time of treatment, duration of treatment, the consumed OHAs or insulin, blood sugar profile, and complications was completed for each patient at the time of first contact.

### 2.9 Data Evaluation and Analysis

Data was entered in SPSS Statistics software for Windows version 17.0. Due to the descriptive nature of the study and moderate sample size, we did not perform a formal test of normality. Descriptive statistics were measured in frequencies and percentages, and continuous data were presented as mean and standard deviation and. Test of association was assessed using the chi‐square test for categorical variables and the independent sample *t*‐test for continuous variables. *P*‐values of ≤ 0.05 were taken as significant.

## 3 Results

A total of 133 patients were enrolled in the study, out of which 65 (49%) were male, and 68(51%) were female. The mean age of patients was 31.4 ± 5.4 years. Most of the patients (82%, *n* = 109) were symptomatic at the time of diagnosis. The most common manifestation was polyuria (68%, *n* = 91), followed by fatigue (60%, *n* = 80) and weight loss (50%, *n* = 66). Family history of diabetes was present in 46(34.6%), and the mean duration of diabetes was 5.4 ± 0.4 years. Among 133 patients, 32(24%) were insulin dependent. 96(72.1%) of them were on oral hypoglycemic agents alone. 5(4%) of patients were on diet therapy alone.

The sociodemographic profile and baseline characteristics of the study population are summarized in the Table 1 and Table 2. The baseline laboratory values are summarized in Table 3. The mean HbA1c level and LDL level were 8.0 ± 2.2% and 122.3 ± 4.1 mg/dL, respectively. In our study, almost 45% (*n* = 59) of patients had blood pressure within normal range (i.e., < 120/80). Only 8% (*n* = 11) of patients had blood pressure more than 140/90.

**Table 1:** Socio‐demographic characteristics of patients enrolled (*n* = 133).

Parameters	Categories	*n*	Percentage
**Demographic picture**			
Age (years)	20–24 years 25–29 years 30–34 years 35–39 years	20	15 18 33 34
		24	
		44	
		35	
Gender	Male Female	65 68	49 51
Address	Terai Hill	97 29 7	73 22 5
	Mountain		
Education	With Formal education Without Formal education	118 15	89 11
Personal History	Smoker Alcohol consumer	33 48	25 36
Family History of DM		46	34.6
Insulin therapy		32	24

**Table 2:** Clinical characteristics of patients (*n* = 133).

Parameters	Mean ± SD
Duration of diabetes (in years)	5.4 ± 0.4 years
Physical examination	
BMI (kg/m^2^)	24.9 ± 4.6
Blood pressure (mm Hg) SBP; DBP)	
SBP	118.7 ± 14.4
DBP	73.1 ± 11.2

Abbreviations: BMI, body mass index; DBP, diastolic blood pressure; SBP, systolic blood pressure.

**Table 3:** Baseline Laboratory Values of patients enrolled (*n* = 133).

Parameters	Values (Mean ± SD)
Fasting Blood sugar	184.7 ± 7.6 mg/dL
Postprandial blood sugar	284.8 ± 12.3 mg/dL
HbA1c	8.0 ± 2.2%
Cholesterol	194.7 ± 4.4 mg/dL
LDL	122.3 ± 4.1 mg/dL
HDL	43.7 ± 0.5 mg/dL
Triglycerides	204.3 ± 7.8 mg/dL
Urine albumin	
Nil‐ *n*(%)	81 (75)
1+ *n*(%)	14 (13)
2+ *n*(%)	7 (6.5)
3+ *n*(%)	6 (5.5)

Abbreviations: HDL, high‐density lipoprotein; LDL, low‐density lipoprotein.

69(52%) had complications. Neuropathy (30%), nephropathy (25%), and ketoacidosis (23%) were the common complications.

The Table 4 below depicts the clinical profile of the patients presenting with ketoacidosis. For further analysis, patients were categorized based on the presence or absence of DKA. This grouping was used as a proxy to explore potential differences between phenotypes suggestive of T1DM versus T2DM, recognizing that this is a simplified classification. Using the Chi‐square and *t*‐test where feasible, age of onset, current age, family history, insulin dependent status, and BMI showed statistically significant difference between the two groups (*p* value < 0.05).

**Table 4:** Clinical profile of patients on basis of ketoacidosis (Chi‐square/*t*‐test).

Variables	DKA (*n* = 31)	No DKA (*n* = 102)	*P*‐value
Age of onset of DM (years; mean ± SD)	18.9 ± 4.6	28 ± 4.7	< 0.001
Current age (years; mean ± SD)	26.7 ± 4.8	32.9 ± 4.8	< 0.001
Males [*n*(%)]	13 (42)	51 (43)	0.25
Family history [*n*(%)]	6 (19)	40 (39)	< 0.001
Insulin Dependent [*n*(%)]	24 (77)	8 (8)	< 0.001
BMI(kg/m^2^; mean ± SD)	21.1 ± 3.4	25.8 ± 4.5	< 0.001
Peripheral neuropathy [*n*(%)]	1 (3)	36 (35)	< 0.001
Retinopathy [*n*(%)]	2 (6)	12 (12)	< 0.001
HbA1c (mean ± SD)	7.9 ± 2.0	7.8 ± 2.3	0.82
Total cholesterol (mg/dL; mean ± SEM)	198.2 ± 6.2	192 ± 4.7	0.51
LDL (mg/dL; mean ± SEM)	115.7 ± 8.6	122 ± 3.9	0.46
TG (mg/dL; mean ± SEM)	205.6 ± 13.9	200 ± 7.8	0.74
HDL (mg/dL; mean ± SEM)	43.1 ± 0.9	44.0 ± 0.6	0.45

**Abbreviations:** BMI, body mass index; HDL, high‐density lipoprotein; LDL, low‐density lipoprotein; TG, triglyceride.

## 4 Discussion

Diabetes mellitus is a major public health problem among the young adult population of Nepal [11]. Due to the increasing incidence in young adults and subsequent complications it is causing a greater burden on health resources and the economy of the country. This study offers important insights into the demographic, clinical, and treatment profiles of young adults living with diabetes. In the present study, there were altogether 133 participants with the mean age of patients being 31.4 ± 5.4 years. 49% were male, and 51% were female. This is in contrast to previous studies like Unnikrishnan et al. [6], and Daga et al. [12] where male patients were found to be recruited more; however, in our study, gender distribution was almost equal. It indicates shifting epidemiology or more balanced healthcare access in our settings. Most of the patients (82%) were symptomatic at the time of diagnosis. This is less in contrast to a study conducted by Dhanwal et al. [5], where all 51 subjects (100%) included in the study were symptomatic, but is comparable to that of a study by Daga [12], where 82% had osmotic symptoms.

The most common symptom found was polyuria in 68% cases, followed by fatigue in 60% of cases, whereas in a study conducted by Soni et al. [13] patients were less symptomatic at presentation with polyuria in 47% of cases, fatigue in 40% of cases and polydipsia in 27% of cases. However, in a study conducted by Banerjee et al. [14], osmotic symptoms were present in 64% of cases, which is comparable to our study. In our study, 52% of patients enrolled had complications, whereas in study done by Soni et al. [13], only 23% of patients had developed complications. Ketoacidosis was an important factor contributing to a higher percentage (23%) of complications due to the selection of young adults with diabetes, and many of them were insulin dependent. The major bulk of complications comprised of neurological complications (30%) and nephropathy (25%) in the form of albuminuria or deranged creatinine and superficial infections (23%). Retinopathy was present in 11% of the study population. In the study conducted by Dhanwal et al. [5], 23.5% presented with ketoacidosis, whereas there were no signs of retinopathy, neuropathy, or nephropathy in any of the patients. A study showed [15] 46.60% patients with T1DM presenting with DKA at onset. These findings suggest poor long‐term glycemic control or possibly delayed diagnosis. The high rate of complications in a relatively young cohort is concerning and signals the urgent need for early screening and intervention programs. A higher percentage of peripheral neuropathy was seen in the non‐DKA group. This may be likely explained by longer disease duration, older age of presentation, and higher prevalence of type 2 diabetes.

Microvascular complications were much lower in study done by Soni et al. [13]. Diabetic neuropathy followed by retinopathy was the most common complication seen in patients with T2DM [16]. Such high percentage of microvascular complications in our study might be attributed either to late diagnosis or poor management issues related to the reluctance in use insulin with longer duration of detrimental hyperglycemia. Almost 50% of patients were diagnosed with diabetes within less than 5 years. 22% of people have been diagnosed with diabetes for more than 10 years. Clinically, these findings emphasize the need for heightened vigilance for diabetes in younger individuals, early risk stratification, and timely initiation of appropriate therapy. In resource limited setting like ours, pragmatic markers like DKA at presentation may assist in identifying patients who require early insulin therapy, but we need to acknowledge the inherent limitations of such an approach.

The mean BMI of patients was 24.9 ± 4.6 kg/m^2^. In comparison, the mean BMI was slightly lower in the study conducted by Dhanwal et al. [5], where it was 21 ± 4.5 kg/m^2^, and it was much lower in that of study of Daga et al. [10] (17.7 ± 1.7 kg/m^2^). However, the mean BMI in our study was comparable to the study conducted by Soni et al. [13], where the mean BMI was 23.2 kg/m^2^, and also to the study by Zargar et al. [17], where the mean BMI was > 25 kg/m^2^. BMI was probably higher in our study in comparison to the previous of 3 studies because we had included the population of the higher age group in comparison to those studies, which increased the chances of including more patients with T2DM. It was further influenced by the increasing incidence of T2DM even among young adults due to increased obesity in younger age groups, predominantly sedentary lifestyle, food habits, and environmental changes. These findings reinforce the need for preventive strategies targeting lifestyle modifications even among younger populations. There was a higher prevalence of DKA among younger patients with lower BMI, which may suggest a phenotype closer to insulin‐deficient individuals.

In our study, almost 45% of patients had blood pressure within normal range (i.e., < 120/80 mmHg). Only 8% of patients had blood pressure more than 140/90 mmHg. This is much less in comparison to other studies. In another study [2], they had concomitant hypertension in > 40% of patients enrolled. Similarly, in a study conducted by Soni et al. [13], 23.6% of patients were hypertensive. The discrepancy might be due to the analysis of blood pressure according to different guidelines. Study [2] and others staged hypertension according to JNC‐7 guidelines, whereas we tried to classify according to the basis of necessity of treatment as per JNC‐8 guidelines.

There might be a relationship between hyperglycemia and hypertension. Some studies reported increased hypertension with increased hyperglycemia [18]. The prevalence of hypertension was high in the diagnosed diabetes mellitus and undiagnosed diabetes mellitus groups. The present study showed hypertension among 8% of patients enrolled. A study done at a tertiary center in Nepal, including patients with T2DM, showed hypertension as a co‐morbidity in 69.60% patients [19]. In the present study, the mean HbA1c was 8.0 ± 2.2. This is less in comparison to previous studies with higher values in other respective studies, such as 9.0 ± 2.2 [6], 11.55 ± 3.1 [5], and 10.5 ± 2.8 [12]. The difference in HbA1c level might be attributed to HbA1c level taken at various times of presentation at OPD; not exactly at the time of diagnosis. Still, the suboptimal glycemic control underscores the need for tighter monitoring and early therapeutic interventions.

Positive family history of diabetes was present in 35% of the patients in our study, which is much higher in comparison to a study done by Soni et al. [13], where 8% of cases had positive family history. Only 4.6% of cases had positive family history in the study conducted by Zargar et al. [17], which is further lower. However, in a study done by Daga et al. [12], 26.4% had positive family history in T1DM patients, and in a study done by Unnikrisnan et al. [6], 67% had positive family history in T2DM patients and 22% in T1DM patients, which is comparable with our study.

Regarding the treatment, 24% of the patients recruited were insulin‐dependent. There is a higher number of insulin‐dependent patients since we had included patients of the young adult age group, and there were higher chances of T1DM patients being recruited. Most of the patients were on OHAs alone (71%), with 4% of patients being on diet therapy alone. A study showed 17.80% T2DM patients under an insulin regimen, suggesting poor glycemic control nature [19].

It was a hospital‐based study, so Berkesonian bias couldn't be eliminated. This study was done in tertiary center in eastern part of Nepal so whether the results can be generalized to all population cannot be ascertained. Since our center is a tertiary center, referral bias could not be excluded. Anti‐GAD antibody, C‐peptide, and Urine for microalbumin couldn't be done due to the inability to arrange funds for the costly tests. So, the confirmation of the type of diabetes could not be done. Using ketoacidosis as a proxy for diabetes type is one of the limitations of our study. This approach was adopted due to financial constraints preventing routine immunological testing like anti GAD antibodies, and C‐ peptide level. This may have resulted in misclassification, particularly in patients with ketosis prone type 2 diabetes.

## 5 Conclusion

Based on this study, we discovered that the presence of ketoacidosis is associated with diabetes in younger age patients, and relatively lower BMI. There is a significant number of patients without ketoacidosis, which is used as a rough estimate of T2DM, which helps us consider that T2DM (based on clinical profile) is occurring more in the young adult age group (20‐39 years), though conventionally, T2DM is assumed to be a disease of the older population. These findings should be cautiously interpreted, given the absence of immunological confirmation, especially in low resource settings. The use of DKA as a differentiating marker might offer clinical value in stratifying patients for early insulin initiation and more aggressive management. This highlights the need for context‐appropriate strategies.

## Author Contributions

**Subarna Giri:** data curation, formal analysis, writing – review and editing. **Smile Kharel:** writing – original draft, formal analysis. **Laxmi Shrestha:** writing – original draft. **Robin Maskey:** conceptualization, writing – review and editing; supervision.

## Funding

The authors received no specific funding for this work.

## Conflicts of Interest

The authors declare that there are no conflicts of interest.

## Transparency Statement

The lead author, Subarna Giri, affirms that this manuscript is an honest, accurate, and transparent account of the study being reported; that no important aspects of the study have been omitted; and that any discrepancies from the study as planned (and, if relevant, registered) have been explained.

## Data Availability

The data that support the findings of the present study are available on reasonable request. However, they are not publicly available due to privacy and ethical restrictions. All authors have read and approved the final version of the manuscript. Dinesh Upreti had full access to all of the data in this study and takes complete responsibility for the integrity of the data and the accuracy of the data analysis.
